# Folic Acid-Adorned Curcumin-Loaded Iron Oxide Nanoparticles for Cervical Cancer

**DOI:** 10.1021/acsabm.1c01311

**Published:** 2022-02-24

**Authors:** Marzieh Ramezani Farani, Maryam Azarian, Hamid Heydari Sheikh Hossein, Zohreh Abdolvahabi, Zahra Mohammadi Abgarmi, Arash Moradi, Seyyedeh Maedeh Mousavi, Milad Ashrafizadeh, Pooyan Makvandi, Mohammad Reza Saeb, Navid Rabiee

**Affiliations:** † Toxicology and Diseases Group (TDG), Pharmaceutical Sciences Research Center (PSRC), The Institute of Pharmaceutical Sciences (TIPS), Tehran University of Medical Sciences, 1417614411 Tehran, Iran; ‡ Department of Radiology, Charité - Universitätsmedizin Berlin, Charitéplatz 1, Berlin 10117, Germany; § Department of Biotechnology, Faculty of Biological Sciences and Technology, University of Isfahan, Isfahan 81746-73441, Iran; ∥ Metabolic Diseases Research Center, Research Institute for Prevention of Non-Communicable Diseases, Qazvin University of Medical Sciences, Qazvin 241567, Iran; ⊥ Department of Clinical Biochemistry, Faculty of Medical Science, Tarbiat Modares University, Tehran 1668814811, P.O. Box: 14115-331, Iran; # Department of Medical Biotechnology, National Institute of Genetic Engineering and Biotechnology, Tehran 1668814811, P.O. Box: 14956-161, Iran; ∇ School of Medicine, Bam University of Medical Sciences, Bam 7661771967, Iran; ○ Faculty of Engineering and Natural Sciences, Sabanci University, Orta Mahalle, Üniversite Caddesi No. 27, Orhanlı, Tuzla, Istanbul 34956, Turkey; ◆ Sabanci University Nanotechnology Research and Application Center (SUNUM), Tuzla, Istanbul 34956, Turkey; ¶ Istituto Italiano di Tecnologia, Centre for Materials Interfaces, viale Rinaldo Piaggio 34, 56025 Pontedera, Pisa, Italy; & Department of Polymer Technology, Faculty of Chemistry, Gdańsk University of Technology, G. Narutowicza 11/12 80-233, Gdańsk, 80-233, Poland; ● Department of Physics, Sharif University of Technology, P.O. Box 11155-9161, Tehran, Iran; ◊ School of Engineering, Macquarie University, Sydney, New South Wales 2109, Australia

**Keywords:** cervical cancer therapy, curcumin, iron oxide nanoparticles, MRI, polyglycerol, targeted delivery

## Abstract

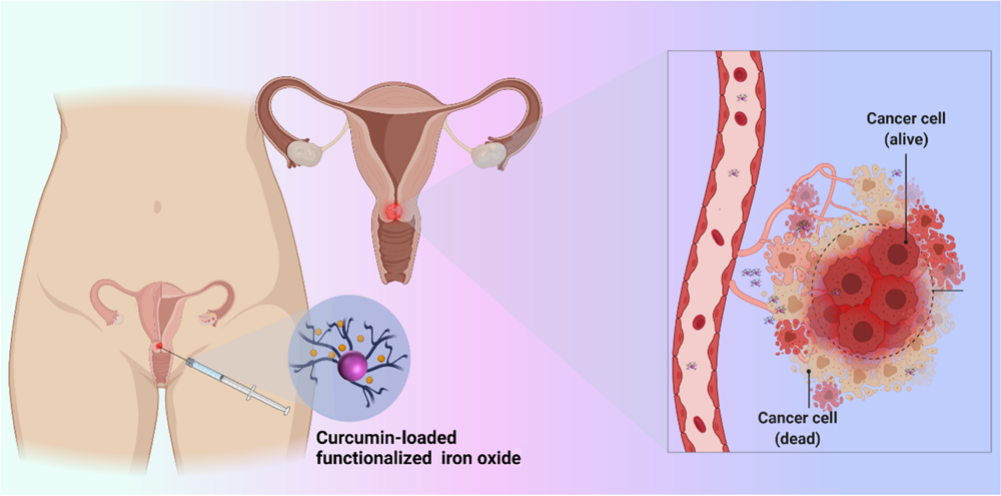

Cancer is a deadly disease that has long plagued humans and has become more prevalent in recent years. The common treatment modalities for this disease have always faced many problems and complications, and this has led to the discovery of strategies for cancer diagnosis and treatment. The use of magnetic nanoparticles in the past two decades has had a significant impact on this. One of the objectives of the present study is to introduce the special properties of these nanoparticles and how they are structured to load and transport drugs to tumors. In this study, iron oxide (Fe_3_O_4_) nanoparticles with 6 nm sizes were coated with hyperbranched polyglycerol (HPG) and folic acid (FA). The functionalized nanoparticles (10–20 nm) were less likely to aggregate compared to non-functionalized nanoparticles. HPG@Fe_3_O_4_ and FA@HPG@Fe_3_O_4_ nanoparticles were compared in drug loading procedures with curcumin. HPG@Fe_3_O_4_ and FA@HPG@Fe_3_O_4_ nanoparticles’ maximal drug-loading capacities were determined to be 82 and 88%, respectively. HeLa cells and mouse L929 fibroblasts treated with nanoparticles took up more FA@HPG@Fe_3_O_4_ nanoparticles than HPG@Fe_3_O_4_ nanoparticles. The FA@HPG@Fe_3_O_4_ nanoparticles produced in the current investigation have potential as anticancer drug delivery systems. For the purpose of diagnosis, incubation of HeLa cells with nanoparticles decreased MRI signal enhancement’s percentage and the largest alteration was observed after incubation with FA@HPG@Fe_3_O_4_ nanoparticles.

## Highlights

- Development of HPG@Fe_3_O_4_ nanoparticles for cervical cancer diagnosis and therapy
- Increasing the cell selectivity by functionalization of nanoparticles with folic acid (FA)
- High loading efficiency and effective delivery of curcumin
- Reducing viability of HeLa cells and contribution to diagnosis via MRI contrast

## 1 Introduction

The fourth most common malignant gynecological tumor in women is cervical cancer. In 2018, cervical cancer caused 311,000 deaths from 570,000 diagnosed cases.^1^ One-half of the number of women that died from cervical cancer were aged ≤58 years, and with the women with ages of 20–39 years, cervical cancer is shown to be the second leading cause of death. Overall, the incidence rate of cervical cancer has demonstrated a decrease in recent years, but distant-stage disease and cervical adenocarcinoma are a threat to the life of many young women around the world, and these cannot often be diagnosed by cytology.^2^ Cervical cancer is more common in developing countries compared to developed countries.^3^ In addition to cytology for detection of cervical cancer, there have been preventative measures in which vaccination against human papillomavirus is the most important.^4^ However, cervical cancer is still a leading cause of death among women and new therapeutic strategies should be designed for its treatment.^5^ Nanotechnological approaches including the use of microfluidic devices, high-gravity techniques, microporous smart nanostructures, green methodologies, and combinations would be a great help in order to improve the efficiency, reduce the cost of methods, increase the greenness factors, and also reduce the time of drug/method discovery.^6−9^

Curcumin is a naturally occurring compound derived from rhizome of *Curcuma longa*.^10^ As a bioactive compound, curcumin has been utilized in treatment of various diseases due to its pharmacological and biological activities including anti-oxidant, anti-inflammatory, anti-diabetic, neuroprotective, and more importantly, anti-cancer.^11,12^ Curcumin has been administered for treatment of cervical cancer. Curcumin administration (13 μM) stimulates DNA damage in HeLa cells and promotes translocation of p53 and H2A.Xser140 with a route from the cytoplasm to nucleus.^13^ Curcumin induces cell cycle arrest at G2/M phases and enhances reactive oxygen species (ROS) generation to stimulate apoptosis and autophagy in reducing the viability of cervical cancer cells.^14^ For potentiating the anti-tumor activity of curcumin, its combination with emodin has been administered to inhibit Wnt/β-catenin signaling via TGF-β down-regulation, resulting in reduction in cervical cancer progression.^15^ However, owing to poor bioavailability and rapid metabolism of curcumin, this phytochemical is not capable of completely eradicating tumor cells. Therefore, nanoformulations have been developed for its delivery and boosting cancer suppression.^16^

The nanoparticle-based precision medicine leads to formulation of new types of early diagnosis/detection and treatment methods.^17−20^ In this regard, the size,^21,22^ electronic properties,^23,24^ zeta potential,^25^ surface functional groups,^26−29^ porosity,^30−32^ and also the potential interactions^33^ affect the possible biomedical applications. In addition, all of these physicochemical parameters should be optimized before addressing the critical issues in the nanomedicine field, in which these optimizations showed critical feedbacks in the diagnosis and treatment perspectives of SARS-CoV-2 as well.^34−36^ Iron oxide nanoparticles (IONPs) are extensively utilized for diagnostic aims and MRI contrast due to their superparamagnetic tools, and they show various beneficial characteristics including high solubility and biocompatibility. The IONPs demonstrate biodegradability, they are distributed in different organs of the body, and no long-term toxicity has been reported.^37^ A recent experiment revealed the role of folic acid (FA)-modified IONPs in diagnosis of breast cancer.^38^ Notably, IONPs can be used for curcumin delivery in cancer treatment. The curcumin-loaded IONPs can suppress SHH signaling and reduce stemness of pancreatic cancer cells to mediate their sensitivity to gemcitabine chemotherapy.^39^ Different studies have evaluated the biocompatibility of curcumin-loaded IONPs, and it was found that these nanostructures are distributed in various organs such as the liver, spleen, and brain.^40^ They do not induce a significant increase in enzyme levels the in kidney and liver; therefore, they can be utilized as promising therapeutic and diagnostic agents.^41^

Different nanosystems of iron oxide have been developed in order to optimize the drug delivery process, one of which is the polymer nanosystem.^42^ Applying biocompatible polymers, including hyperbranched polyglycerol (HPG), in the coating procedure improves the hydrophilicity and facile attachment of targeting agents on the polymer’s surface. HPG is recognized as a favorable new type of polymer, associated with a biocompatible polyether scaffold, well-defined dendrimer-like architecture, and abundant functional end groups.^43^ HPG is utilized in the current research in order to coat IONPs prior to incubation with or without FA; FA is under consideration as a targeting agent in view of the known overexpression of FA receptors on some cancerous cells.^44,45^ Folate binding protein is known as a glycosylphosphatidylinositol (GPI)-anchored cell surface receptor of folate. FA receptors are observed in small amounts on normal epithelial cell surfaces in various organs, including the kidney, thyroid, lungs, and brain. Of note, they are observed with a high expression ratio in several human tumors. This overabundance of FA receptors on tumor cells might be associated with FA’s vital role in cancer cell proliferation.^46^ Recent studies showed that the presence of specific biomarkers on the surface of the nanocarriers would lead to increasing the efficient targeting; however, (over) expression of different types of genes should be investigated before any attempts on this context.^47−49^ Also, the iron-based nanoparticles/nanomaterials could be able to mimic the electron transfer between the cellular membranes; therefore, they are completely favorable to make strong interactions with the cells.^50−52^

It is proposed that anticancer drugs bound to HPG-coated magnetic IONPs that have FA attached might achieve increased bioavailability in tumor tissues as a result of targeting via FA receptors.^53,54^ A number of studies have applied natural and synthetic polymers to coat IONPs. Huang et al. utilized polyethylene glycol-coated nanoparticles in the doxorubicin transfer process.^55^ Akbarzadeh et al. evaluated nanoparticles that were encapsulated into poly(d,l-lactic-*co*-glycolic acid), poly (ethylene glycol), and (PLGA-PEG) nanoparticles.^56^

Appropriate polymer selection directly depends on the biocompatibility, hydrophilicity, and non-adsorption properties of the given protein. HPG is defined as a type of hydrophilic polyether having unique characteristics including high biocompatibility, minimal toxic effects, and easy synthesis. HPG also contains several terminal hydroxyl groups that are available for the binding of targeted ligands.^57^ The synthesis of Fe_3_O_4_ nanoparticles using the polyol method was carried out in the current study, and then HPG was added by the anionic ring-opening polymerization method. HPG coating’s impact on Fe_3_O_4_ nanoparticles’ biocompatibility enhancement was measured. A covalent bond between FA and polyglycerol’s terminal hydroxyl groups was then generated. The procedure of loading curcumin onto the resultant FA-coupled nanoparticles and consequent evaluation of related loading and releasing efficiencies were then carried out using FT-IR, TEM, DLS, CHNS, and TGA analyses. The cytotoxicity of nanoparticles on HeLa cell lines was evaluated, and mouse L929 fibroblasts as normal cells were used. Finally, the potential of nanoparticles in diagnosis was evaluated by MRI contrast.

## 2 Materials and Methods

### 2.1 Synthesis and Surface Functionalization of Fe_3_O_4_

**Figure 1:**
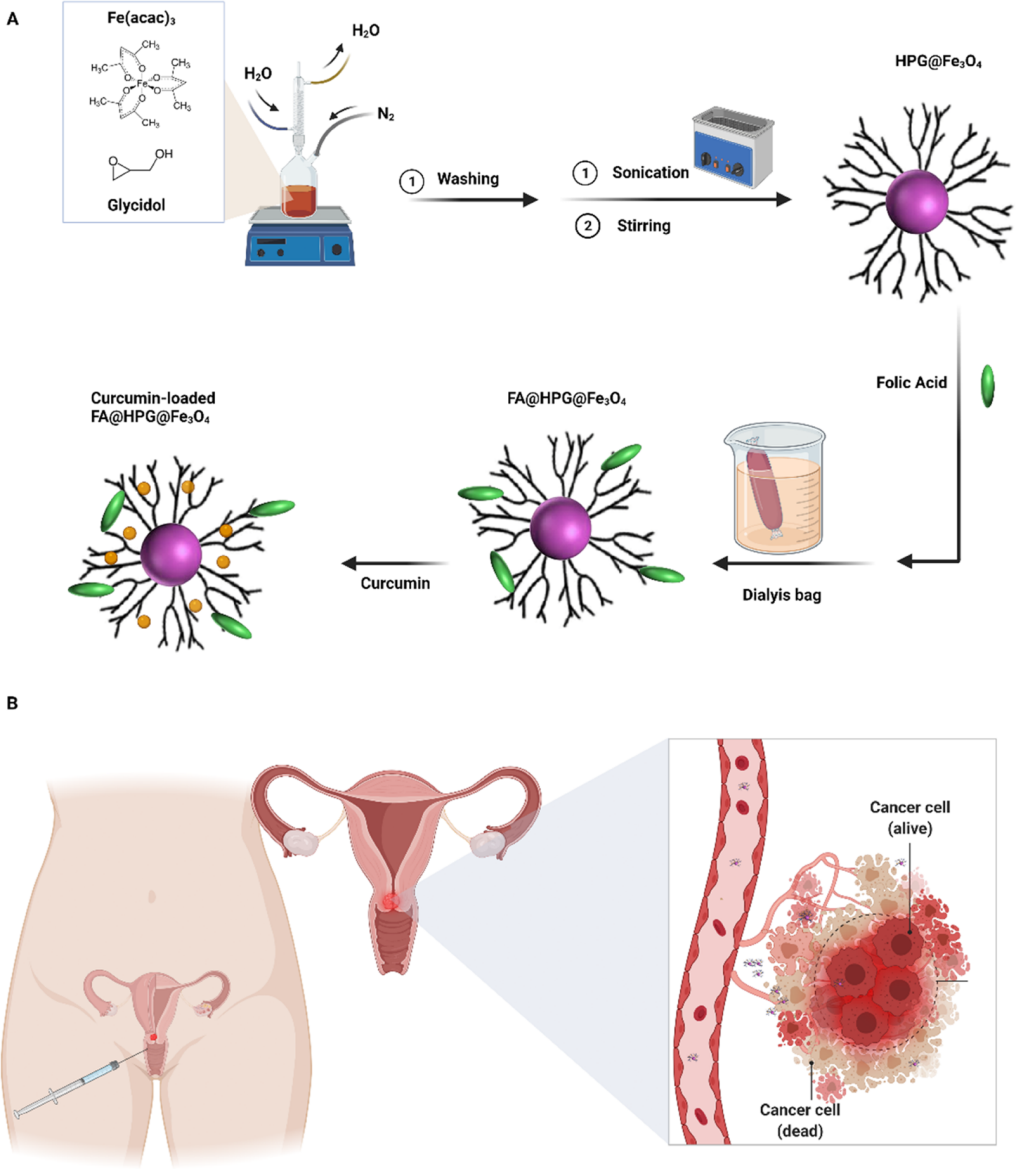
(A) Synthesis of HPG@Fe_3_O_4_ and FA@ HPG@Fe_3_O_4_ nanoparticles. (B) Schematic illustration of potential applications of folate-targeted for cervical cancer treatment.

IONPs were synthesized by a polyol method and coated with polyglycerol-branched polymers using the looping mechanism. A magnetic stirrer was used to combine 0.53 g of iron(III) acetylacetonate (Fe(acac)_3_) and 30 mL of triethylene glycol) TREG) components. This mixture was gradually heated until the boiling point was reached (285 °C). It was kept in an atmosphere of N_2_ for about 30 min under reflux conditions since the heating process was completed within a 3 h period. A black homogeneous solution was obtained. After reaching room temperature, 20 mL of ethyl acetate was added. A neodymium magnet was applied in order to collect the black precipitate. The black precipitate containing uncoated IONPs was washed with ethyl acetate three times in order to remove the additional TREG and was dried under vacuum to a powder form. HPG coating of the nanoparticles was initiated by the addition of 4 mL of glycidol to 30 mg of Fe_3_O_4_ nanoparticles and a 1 h sonication process. The black compound was mixed using a magnetic stirring process for 20 h at 140 °C in an atmosphere of N_2_. Then, the black gel was combined with 15 mL of deionized (DI) water in an ultrasonic bath after reaching room temperature. After exposing this solution to a neodymium magnet, the HPG-coated IONPs (HPG@Fe_3_O_4_) were collected and washed with DI water three times and then dried in a vacuum to obtain a black solid. FA was attached to the HPG-coated nanoparticles at concentrations of either 5, 25, or 50% FA. A combination of 5 mg of FA and 100 mg of HPG@Fe_3_O_4_ was dissolved in 5 mL of dimethyl sulfoxide (DMSO) to form 5% FA-HPG@Fe_3_O_4_ (FA-targeted nanoparticles). A total of 1.53 mg of 4-dimethylaminopyridine (DMAP) and 2.955 mg of ethyl acetate and *N*,*N*′-dicyclohexylcarbodiimide (DCC) were added to HPG@Fe_3_O_4_ solution as a catalyst and binder, respectively. The heating process took place over 36 h to reach the temperature of 50 °C. The solution was then dialyzed in a 12 kDa molecular weight cutoff dialysis bag to remove the remaining portion of DCC, DMAP, and FA. The retained FA@HPG@Fe_3_O_4_ was dried in a freeze dryer.^58,59^ FA@HPG@Fe_3_O_4_ nanoparticles prepared with 25 and 50% FA were generated using the same procedure (Figure 1).

### 2.2 Curcumin Loading onto HPG@Fe_3_O_4_ and FA@HPG@Fe_3_O_4_

Nanoparticles and curcumin were incubated for 24 h at 4 °C and then placed in a shaker at three ratios (1:1, 2:1, and 1:2) with the purpose of loading the curcumin on the nanoparticles. The samples were centrifuged at 4000 rpm for 5 min after 12 h to precipitate the curcumin associated with the nanoparticles.^60,61^ The loading efficiency of drug can be calculated using the following equation:
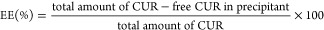
1

### 2.3 Curcumin Release from HPG@Fe_3_O_4_ and FA@HPG@Fe_3_O_4_

Curcumin release from HPG@Fe_3_O_4_ and FA@HPG@Fe_3_O_4_ nanoparticles in phosphate-buffered saline (PBS) at 37 °C was measured during an 8 day period. The nanoparticles with bound curcumin were kept in a dialysis bag (MWCO: 12 kDa) containing a PBS solution for various lengths of time and then centrifuged to determine curcumin release.^62,63^ The concentration of released curcumin was measured by absorbance at 450 nm using UV–Vis. The following equation is used in order to calculate the drug release ratio at the initial curcumin level:
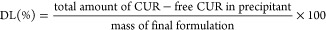
2

### 2.4 *In Vitro* Cytotoxicity Assay

HeLa cells and mouse L929 fibroblasts were incubated with curcumin (free drug) and nanoparticle-loaded curcumin for 24, 48, and 72 h time intervals. Cytotoxicity was measured in curcumin-treated cells using the 3-(4,5-dimethyl-2-thiazolyl)-2,5-diphenyl-2*H*-tetrazolium bromide (MTT) assay. Control experiments were carried out using nanoparticle-free medium. Six replications were considered for each experiment. An ELISA reader was utilized in order to read the wells’ optical absorption at 492 nm wavelength.
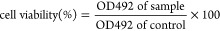
3

### 2.5 Cell Uptake of Nanoparticles

HeLa cells were used to determine HPG@Fe_3_O_4_ or FA@HPG@Fe_3_O_4_ nanoparticle uptake in three 24-well plates at a density of 2000 cells each well. When the cells’ growing phase was achieved after 48 h of incubation at 37 °C, each plate’s medium was replaced with 0.2 mg/mL HPG@Fe_3_O_4_ or FA@HPG@Fe_3_O_4_ for 1, 3, and 7 μL of 5 M HCl applied for 1 h; an MS2000-Skyray inductively coupled plasma-mass spectrometer (ICP-MS) was used to measure the iron concentration released from the cells.^64^

### 2.6 *In Vitro* Magnetic Resonance Imaging (MRI) Experiment

T_2_-weighted signal intensity measurements using a clinical MR scanner (Siemens Magnetom Avanto, 1.5 T) were used to image cells following exposure to nanoparticles. After 24 h of seeding HeLa cells (5 × 10^5^ cells per well) in a 6-well culture plate, various nanocarriers (Fe_3_O_4_, HPG@Fe_3_O_4_, and FA@HPG@Fe_3_O_4_ nanoparticles prepared with 25 wt % FA) were added to cells at a concentration of 0.20 mg/mL. The evaluation of dual targeting impacts on T2-weighted signals was carried out in another experiment through the addition of both HPG@Fe_3_O_4_ and FA@HPG@Fe_3_O_4_ nanoparticles at a concentration of 0.20 mg/mL to cells. After 24 h, the medium was removed, and consequently, cells were washed three times to eliminate free nanoparticles. Afterward, 3 mL of PBS was added to each well and cells were scanned using a 1.5 T MR scanner (TR1000-4000 ms, TE: 15–480 ms).^65,66^

### 2.7 Characterizations

The characterizations were conducted based on our previous publications.^20,26^ A UV–vis spectrometer (Perkin Elmer Lambda 25) was used to record absorbance in the range of 200–800 nm. Fourier transformed infrared spectroscopy (FT-IR) spectrum was obtained using a JASCO FT-IR-460 spectrometer in the range of 400–4000 cm^–1^. The morphology of synthesized nanoparticles/nanomaterials was observed by a field emission scanning electron microscope (FESEM, TESCAN MIRA-3) under an acceleration voltage of 30–250 kV. Transmission electron microscopy (TEM) analysis was done using a TEM JEOL at 300 kV.

### 2.8 Statistical Analysis

MTT data were obtained from a variance analysis of two constant effects followed by Tukey’s test. The level of significance was considered to be 0.05.

## 3 Results and Discussion

### 3.1 Characterization of Nanoparticles

**Figure 2:**
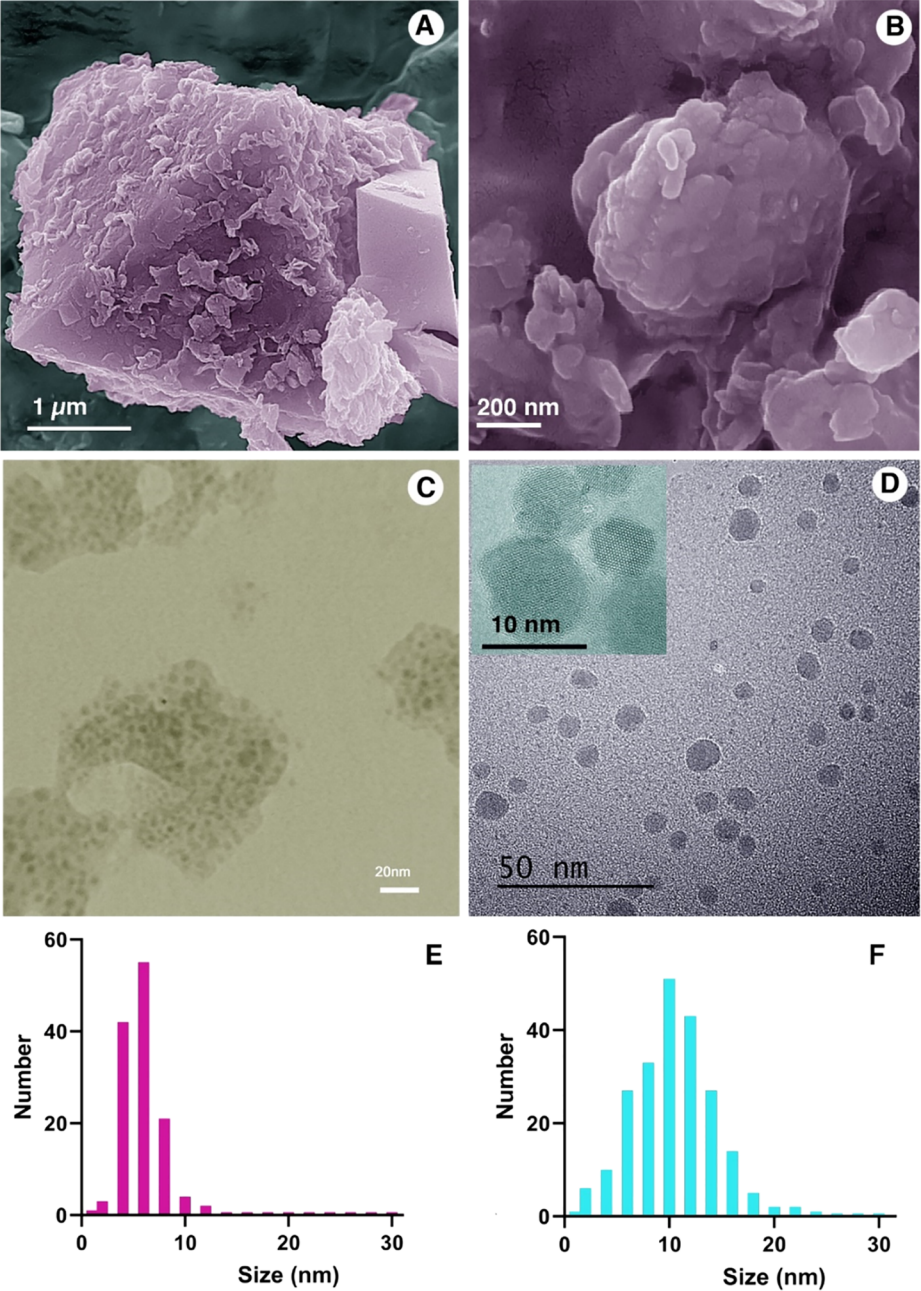
SEM and TEM images of (A and C) Fe_3_O_4_ and (B and D) HPG@Fe_3_O_4_ nanoparticles. Size distribution analysis of (E) Fe_3_O_4_ and (F) HPG@Fe_3_O_4_ nanoparticles.

SEM and TEM images were obtained for IONPs (Figure 2A–D). IONPs prepared without HPG coating showed a uniform size of 5–6 nm (Figure 2E). HPG-coated nanoparticles were somewhat larger, with a size in the range of 10 nm (Figure 2F). HPG-coated nanoparticles displayed improved water dispersibility and limited agglomeration that are of important for the purpose of drug delivery (Figure 2C,D).

**Figure 3:**
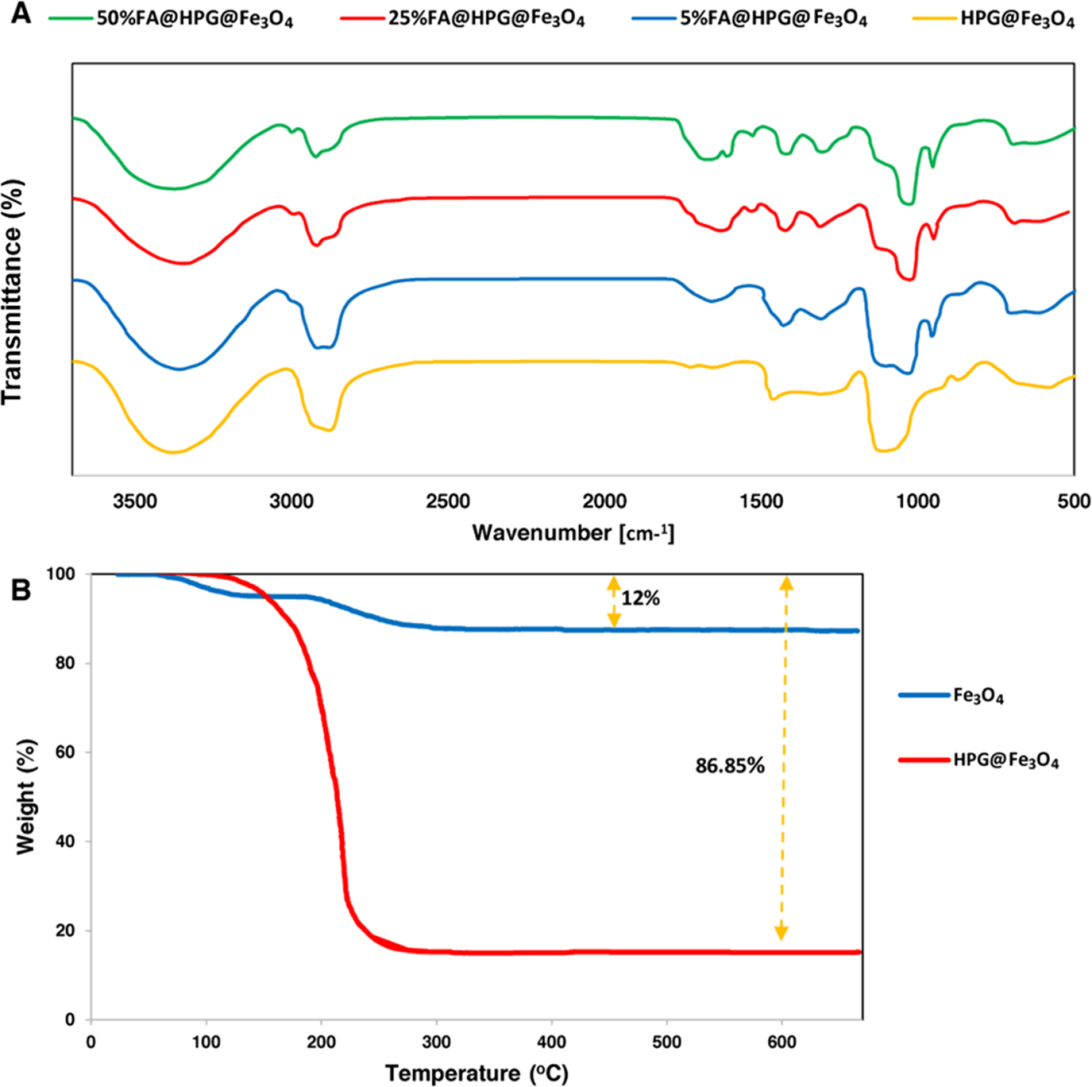
(A) FT-IR test results of HPG-coated nanoparticles treated with 5, 25, and 50% (top trace) folic acid. (B) TGA curves for Fe_3_O_4_ and HPG@Fe_3_O_4_ nanoparticles.

FT-IR analyses were used to characterize HPG-coated nanoparticles treated with 5, 25, and 50% FA. FA-adorned nanoparticles demonstrated a significant increase in the vibrational peak rate of 1100, 2900, and 3300 cm^–1^ for O–H, C–H, and C-O-C bonds, respectively (Figure 3A). An increase in the peak rate of O–H demonstrates the increased hydrophilicity of HPG-coated nanoparticles treated with FA.^67^ The addition of FA to HPG-coated nanoparticles reduced the peak rate of 1100 cm^–1^. Since the overlap between the primary amines of FA and hydroxyl group makes them invisible,^68^ differences are observed in the 3400–3500 and 1560–1640 bands. The peak rate at 1650 cm^–1^ represents the C=O amide group, while the peak rate at 1700 cm^–1^ represents the ester. FA-targeted samples with the peak rates of 1400 and 1620 cm^–1^ belong to pethidine ring and amino-benzoic acid motif, respectively. The peak rate of the C=C bond in six-carbon rings treated with FA was observed in the 1640 band. They could be regarded as the increased peak rates of cover samples.^69^

Figure 3B shows the thermal behavior patterns of Fe_3_O_4_ and HPG@Fe_3_O_4_ nanoparticles. Fe_3_O_4_ nanoparticles show a weight reduction of 12% at elevated temperatures, in accordance with the removal of TREG (i.e., 8%) by the weight ratio of Fe_3_O_4_:TREG (11.56:1). Weight reduction was continued until the temperature of 285 °C was reached (the boiling point of TREG) and was not changed until heating to 700 °C. The results of FT-IR (Figure 3A) and elemental analysis, which confirm the existence of organic molecules of TREG on the Fe_3_O_4_ surface, imply that the magnetic core of the Fe_3_O_4_ nanoparticles is stable despite reaching high temperatures. TGA of HPG@Fe_3_O_4_ nanoparticles showed a larger weight reduction at elevated temperatures (i.e., 86.85%), related to the surface polymer degradation.^70,71^

Table 1 provides a list of results of the elemental analysis of HPG-coated and uncoated Fe_3_O_4_ nanoparticles. It can be observed that 5.85 and 1.17% of particles’ weights are, respectively, related to C and H elements. Since Fe_3_O_4_ and surface-adsorbed TREGs (C_6_H_14_O_4_) constitute Fe_3_O_4_ nanoparticles, it could be concluded that the remaining weight% in the elemental analysis (except C and H), i.e., 92.98%, is attributed to Fe and O (from TREG and Fe_3_O_4_). Thus, weights% of Fe and O are, respectively, determined to be 63.75 and 28.35%. The number ratio of compounds is determined to be C (6):Fe (23):O (34) based on considering the mentioned weight% ratio. Therefore, 7.5:1 and 11.56:1 are, respectively, determined for Fe_3_O_4_:TREG’s number and weight ratio. However, the existence of such a small amount of TREG, which is obtained from the elemental analysis results, supports the conclusions of FT-IR analysis. It should be noted that weights% of Fe_3_O_4_ nanoparticles’ C and H atoms calculated after performing the process of HPG grafting are, respectively, determined to be 31.11 and 10.07 (Table 1). The C:(Fe + O) ratio is determined to be 31.11:58.82. In this case, C and H are attributed to PG, while TREG and O are attributed to PG, TREG, and Fe_3_O_4_. The core of HPG@Fe_3_O_4_ is made from Fe_3_O_4_, while its shell consists of both TREG and PG. Therefore, it can be concluded that the Fe_3_O_4_:TREG:PG’s weight% ratio is 35.98:4.9:59.12, while HPG@Fe_3_O_4_’s weight ratio of the core:shell is 35.98:64.02.

**Table 1:** Elemental Analysis of Fe_3_O_4_ and HPG@Fe_3_O_4_^a^

sample	C (wt %) ± SD	H (wt %) ± SD	N (wt %) ± SD	S (wt %) ± SD
Fe_3_O_4_	5.85 ± 0.00	1.17 ± 0.00	0.00 ± 0.00	0.00 ± 0.00
HPG@Fe_3_O_4_	32.33 ± 0.12	12.17 ± 0.63	0.00 ± 0.00	0.00 ± 0.00

a Data represented as mean ± SD (*n* = 3).

### 3.2 Curcumin Loading and Release

**Figure 4:**
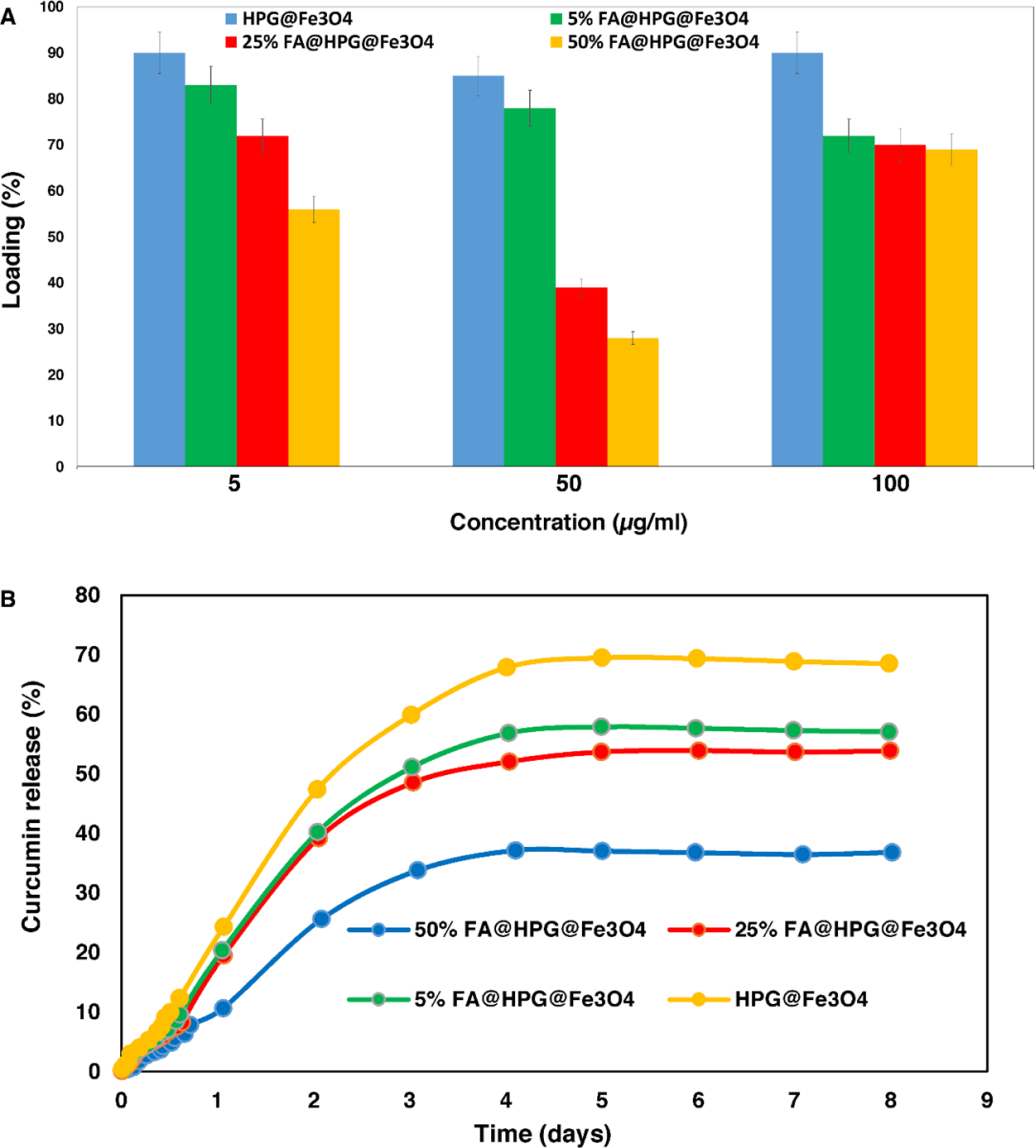
(A) Diagram of loading efficiency for encapsulation of curcumin on 5, 25, and 50% folic acid-coated nanoparticles. (B) *In vitro* release curve of curcumin release from polyglycerol-coated nanoparticles and 5, 25, and 50% folic acid-targeted nanoparticles.

The profiles of curcumin loading and release from HPG@Fe_3_O_4_ or FA@HPG@Fe_3_O_4_ nanoparticles are shown in Figure 4A, respectively. Curcumin loading was greatest in HPG@Fe_3_O_4_ nanoparticles lacking any FA (85–90% loading), as shown in Figure 4A. FA@HPG@Fe_3_O_4_ nanoparticles that had been prepared using 5% FA showed more curcumin loading than nanoparticles prepared with 25 or 50% FA. The data are consistent with the hypothesis that FA attachment to the nanoparticles impairs the ability of curcumin to permeate into the nanoparticles.

The rate of curcumin release from HPG@Fe_3_O_4_ or FA@HPG@Fe_3_O_4_ nanoparticles is shown in Figure 4B. HPG@Fe_3_O_4_ nanoparticles release a higher percentage of their bound curcumin (75%) than FA@HPG@Fe_3_O_4_ nanoparticles (45–60%) *in vitro*. When increasing the concentrations of FA (5, 25, and 50% FA) used to prepare FA@HPG@Fe_3_O_4_ nanoparticles, curcumin was released more completely when 5% FA was used and released least effectively when 50% FA was used.

### 3.3 Cultured Cell Viability Following Exposure to Curcumin

**Figure 5:**
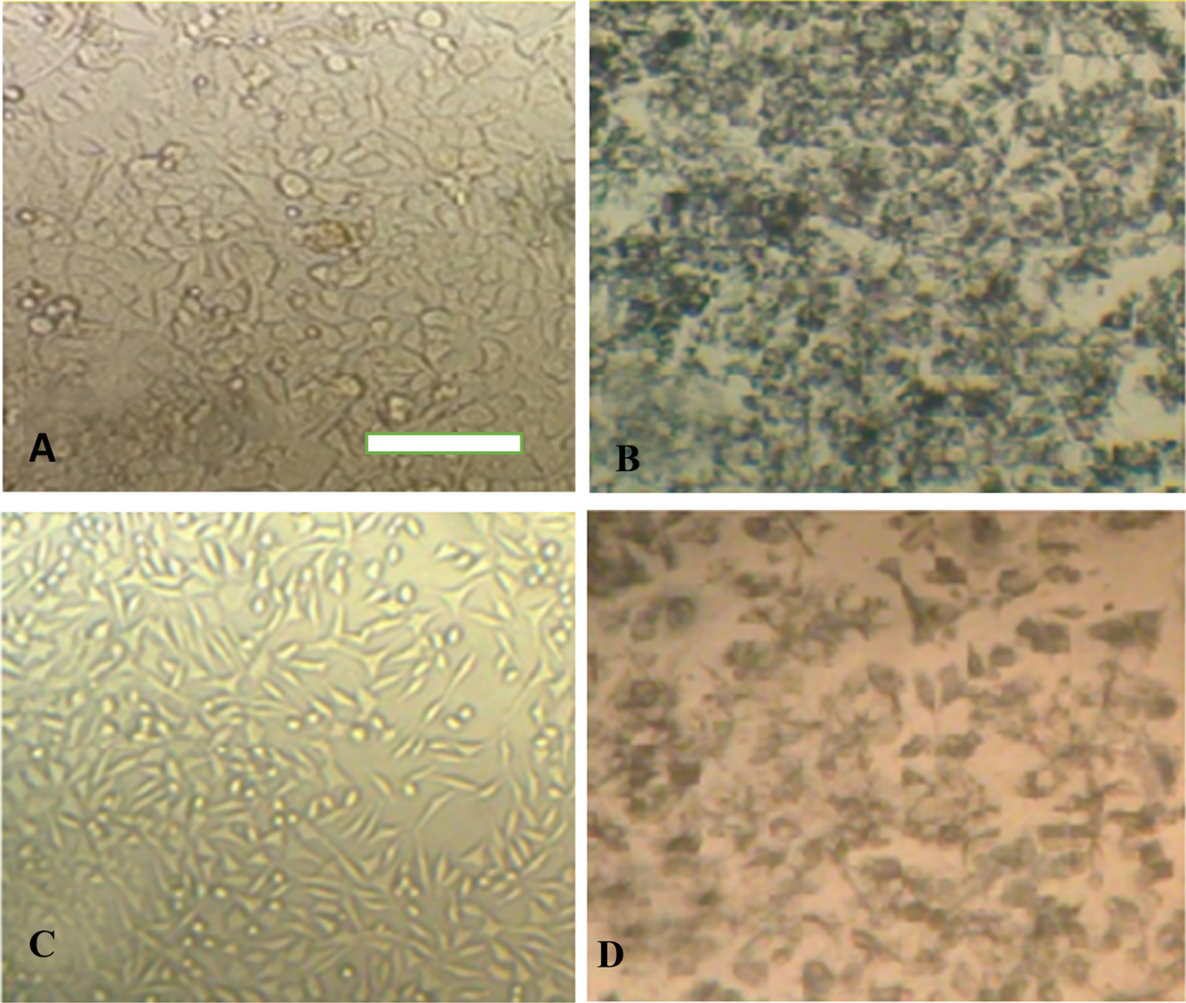
HeLa cell line’s optical microscopy images related to (A) before and (B) after MTT treatment. L929 cell line’s optical microscopy images related to (C) before and after (D) MTT. The scale bar is 50 μm.

The impact of curcumin (free drug) and curcumin-loaded nanoparticles was evaluated *in vitro* in HeLa cells and mouse L929 fibroblasts. Figure 5 depicts Hela cells (A and B) and L929 cells (C and D) before and after incubation with MTT reagents. The morphology of cells has not changed after exposure to nanoparticles. However, nanoparticles have negatively affected the cell wall that impairs electrostatic interactions among cells and leads to aggregation of cells that is observed for both normal and cancer cells (Figure 5B,D).

**Figure 6:**
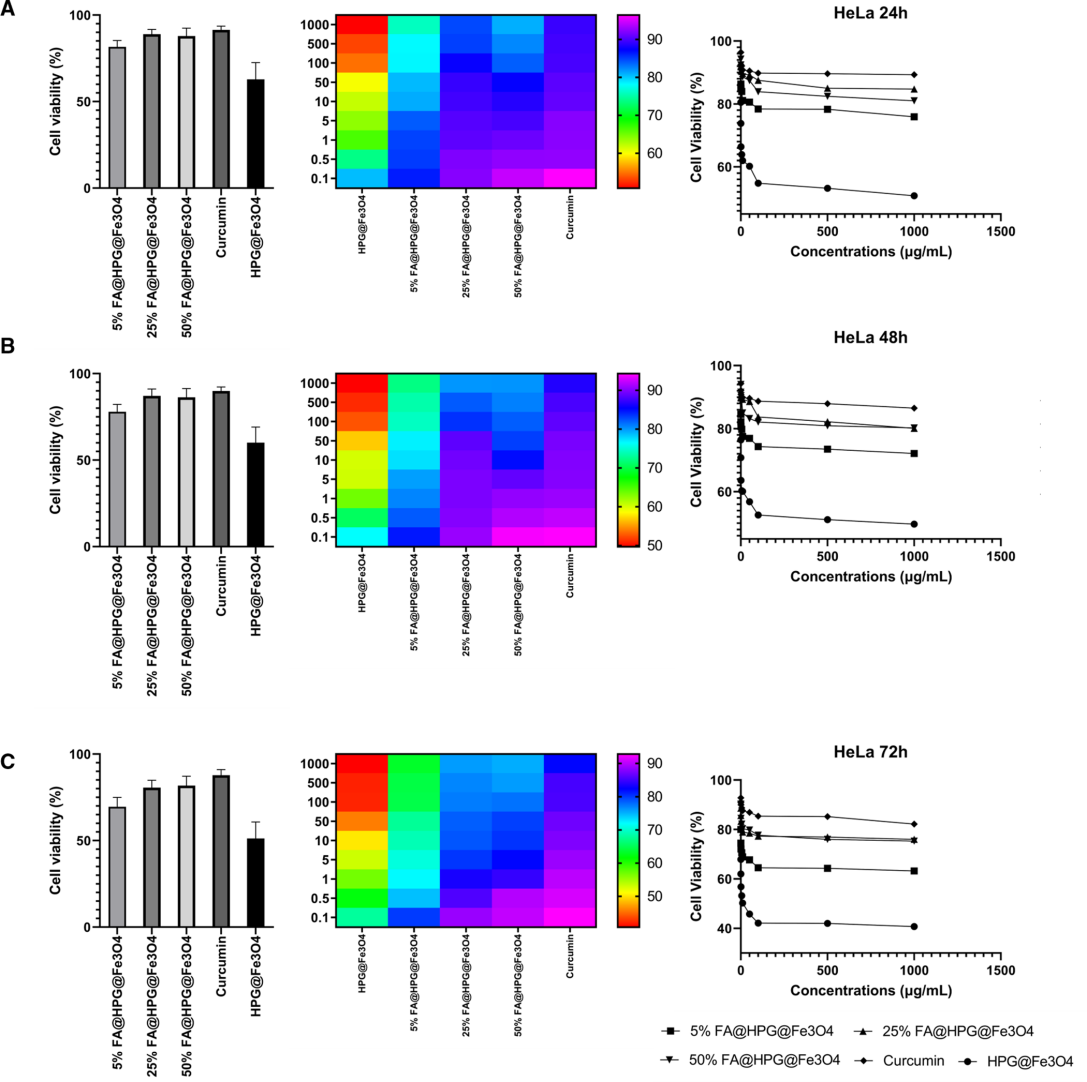
Viability of HeLa cells after exposure to nanoparticles for (A) 24, (B) 48, and (C) 72 h treatment times. Nanoparticles exert their cytotoxicity in a time- and concentration-dependent manner to reduce viability of cancer cells. The lowest viability is observed after 72 h and exposure to HGP@Fe_3_O_4_ nanoparticles.

**Figure 7:**
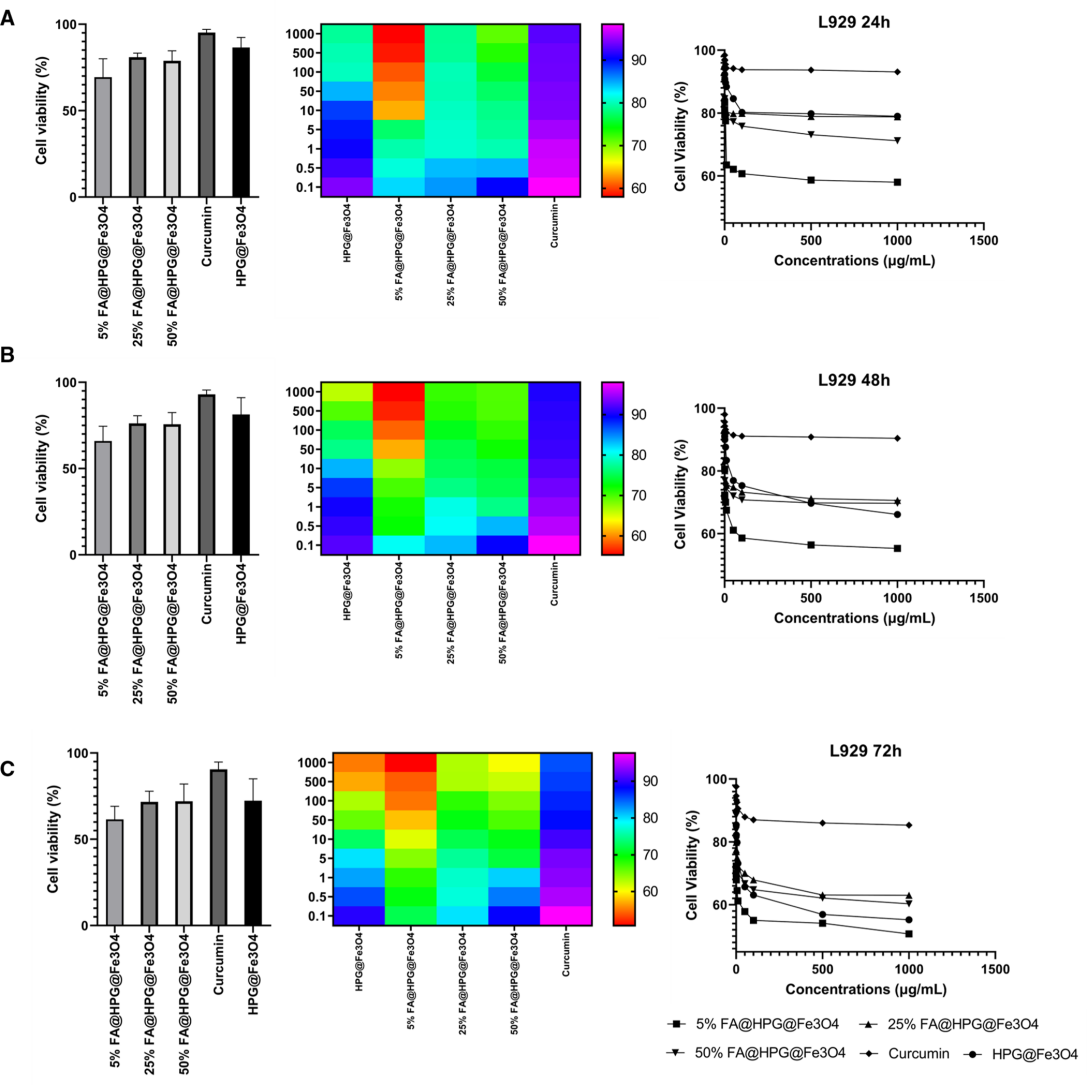
Toxicity evaluation of nanoparticles on L929 fibroblasts as normal cells after (A) 24, (B) 48, and (C) 72 h treatment times. Based on the results, they demonstrate partial toxicity toward normal cells and their overall biocompatibility is promising.

The cell viability data presented in Figure 6 (HeLa cells) and Figure 7 (L929 cells) indicate that curcumin-loaded HPG@Fe_3_O_4_ nanoparticles did cause considerable cell toxicity to tumor cells (Figure 6), but they showed low and negligible toxicity to normal cells (Figure 7). Furthermore, the toxicity toward cancer cells is time-dependent and more toxicity is observed after 72 h compared to 24 h on HeLa cells (Figure 6). HeLa cells treated with curcumin-loaded FA@HPG@Fe_3_O_4_ nanoparticles showed a higher toxicity than the same cells incubated with curcumin-loaded HPG@Fe_3_O_4_ nanoparticles. Therefore, functionalization of nanoparticles with FA can promote their selectivity toward cancer cells that is of interest for reducing cancer cell viability (Figure 7). In mouse fibroblast L929 cells, incubation with curcumin-loaded FA@HPG@Fe_3_O_4_ nanoparticles had a similar effect, with cell viability decreased most when FA@HPG@Fe_3_O_4_ nanoparticles prepared with 5% FA were employed. The data in Figures 6 and 7 provide evidence that curcumin-loaded FA@HPG@Fe_3_O_4_ nanoparticles are more cytotoxic than curcumin-loaded HPG@Fe_3_O_4_ nanoparticles in both cultured cell lines. In addition, as shown in Figures 6 and 7, the cell viability data were generated when HeLa cells (a) and L929 cells (b) were incubated with free curcumin. It is noteworthy that both cell lines were influenced by free curcumin, but with a distinct time profile from the data generated using curcumin-loaded HPG@Fe_3_O_4_ or FA@HPG@Fe_3_O_4_ nanoparticles. Free curcumin was cytotoxic to both HeLa cells and L929 cells in a 24 h or 48 h incubation, but both cell lines appeared to recover from the cytotoxic effects of free curcumin after 72 h. In contrast, the cytotoxic action of curcumin-loaded onto either HPG@Fe_3_O_4_ nanoparticles or FA@HPG@Fe_3_O_4_ nanoparticles was observed most strongly after 72 h incubation.

### 3.4 HeLa Cell Uptake of HPG@Fe_3_O_4_ or FA@HPG@Fe_3_O_4_ Nanoparticles

**Figure 8:**
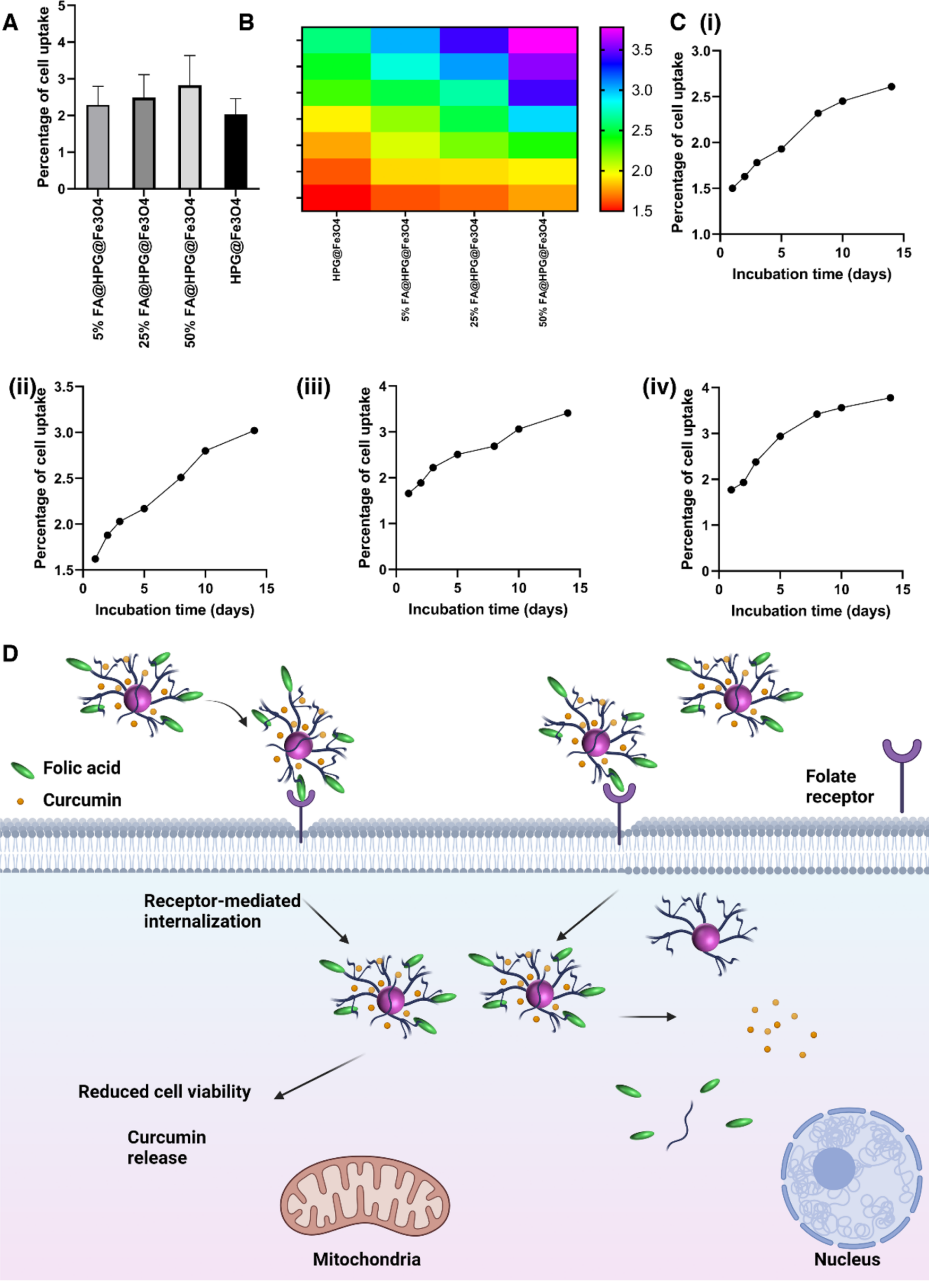
FA@HPG@Fe_3_O_4_ uptake through HeLa cells. (A–C) Cellular uptake of nanoparticles by HeLa cells. (D) Schematic representation of FA-adorned HPG IONPs in internalizing in cells and release of curcumin for cervical cancer therapy.

One of the challenges in cancer therapy is low internalization of anti-cancer agents in tumor cells. Furthermore, anti-tumor compounds, especially plant derived-natural compounds such as curcumin, suffer from poor bioavailability and use of nanocarriers improves its therapeutic index via elevating tumor cell internalization.^72^ The aim of the current work is to enhance cancer cell internalization of curcumin using FA-adorned HPG@Fe_3_O_4_ nanoparticles. HeLa cells were incubated with HPG@Fe_3_O_4_ or FA@HPG@Fe_3_O_4_ nanoparticles for 5, 10, and 15 days, and nanoparticle uptake was measured following acid lysis of the cells (Figure 8A–C). An MS2000-Skyray inductively coupled plasma-mass spectrometer (ICP-MS) was used to measure the iron concentration release from the cells.^67,73^ Cellular uptake ranged from 1.5 to 1.7% for HPG@Fe_3_O_4_ nanoparticles. Cellular uptake ranged from 1.8 to 2.2% for FA@HPG@Fe_3_O_4_ nanoparticles. Therefore, it appears that surface modification of HPG@Fe_3_O_4_ nanoparticles with FA promotes their internalization in cervical cancer cells and this property is of importance for effective cancer therapy. Figure 8D shows the internalization of FA-adorned HGP@Fe_3_O_4_ nanoparticles into cervical cancer cells by binding to the folate receptor.

### 3.5 *In Vitro* MRI of HeLa Cells Incubated with HPG@Fe_3_O_4_, FA@HPG@Fe_3_O_4_, and Fe_3_O_4_ Nanoparticles

**Figure 9:**
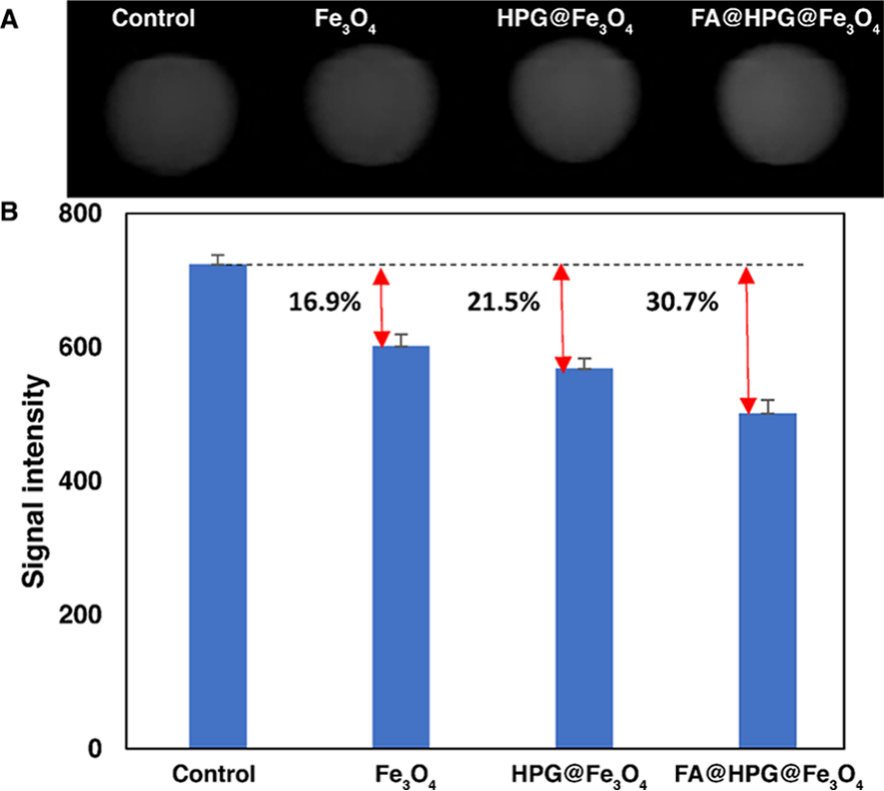
(A) T2-weighted MRI phantom images of HeLa cells after incubation with nanocarriers at a concentration of 0.2 mg/mL (a, left) FA@HPG@Fe_3_O_4_ (25% w: w FA:polymer), (b) HPG@Fe_3_O_4_, (c) control sample, (d, right) Fe_3_O_4_ nanoparticles. (B) Signal intensity and increase in *in vitro* T2-weighted MRI.

The T2-weighted MRI phantom images of HeLa cells incubated with HPG@Fe_3_O_4_, FA@HPG@Fe_3_O_4_, and Fe_3_O_4_ nanoparticles for 24 h can be observed in (Figure 9), and quantitative data are shown in Figure 9. Applying eq 4, the post-incubation MRI images of the cells achieved through using IONPs are calculated:
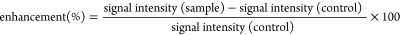
4

The results obtained from the increase in contrasts between various types of nanoparticles are provided in Figure 9. A reduction of MRI signal enhancement’s percentage was observed in HeLa cells incubated with each type of nanoparticle, with the largest change observed in cells incubated with FA@HPG@Fe_3_O_4_ nanoparticles, with 12% targeting ratio (−30.7%). These data are consistent with HeLa cell uptake and viability findings (Figure 9). Based on the literature,^74^ this type of hyperbranched polymer-coated Fe_3_O_4_ nanoparticle is promising due to the trends in the bio-magnetic properties and the slopes between the signal intensities. Fe_3_O_4_ nanoparticles have attracted considerable attention due to their significant bio-magnetic properties, but their interactions to the physiological microenvironments and the possible aggregations/agglomerations inhibit/limit their applications. Therefore, using a hyperbranched bioactive and bioavailable polymers could reduce these limitations and increase the constructive interactions.

## 4 Conclusions and Remarks

There are novel targeted nanodrugs and delivery systems associated with the ability of increasing the anticancer drugs’ loading and delivery efficiency that can activate the drug-release mechanism. Several investigations were carried out with the purpose of designing and forming anticancer drug-carrier nanosystems.^75,76^ The current study describes the synthesis and characterization of small and stable magnetic IONPs, with narrow size distribution, and complexed with HPG hydroxyl groups using the polyol method. The HPG grafting is performed to produce a physiologically stable nanoscale delivery system with high water solubility. FT-IR, TGA, and CHNS elemental analysis confirmed the Fe_3_O_4_, HPG@Fe_3_O_4_, and FA@HPG@Fe_3_O_4_ syntheses, and the nanoparticles’ small size was verified using size analysis. A considerable enhancement of drug aqueous solubility was achieved as a result of the existence of a weak permeation of hydrophobic–hydrophobic linkage into the polyglycerol’s ether backbone. Curcumin loading experiments with the nanoarchitectures confirmed its consequent *in vitro* release kinetics. The MTT assay revealed the high cytotoxicity of curcumin-loaded FA-adorned HPG@Fe_3_O_4_ nanoparticles on HeLa cells and reduction in viability of cancer cells after 24, 48, and 72 h. FA@HPG@Fe_3_O_4_ nanoparticles were able to increase the T2-weighted signal intensity during the MRI process. The addition of FA to the poly-hydroxylated HPG@Fe_3_O_4_ nanoparticles did increase nanoparticles’ cellular uptake, which leads to the enhancement of the nanocarrier’s therapeutic potential. The FA@HPG@Fe_3_O_4_ nanoparticles can bind and release curcumin, and potentially other candidate drugs, for cancer diagnosis and therapy. The MRI test revealed the role of nanostructures in diagnosis of cervical cancer cells; hence, the nanoparticles developed in the current work are promising candidates for treatment and diagnosis of cervical cancer.

## Abbreviations

IONPs: iron oxide nanoparticles
FA: folic acid
HPG: hyperbranched polyglycerol
GPI: glycophosphatidylinositol
DI: deionized
DMSO: dimethyl sulfoxide
DMAP: 4-dimethylaminopyridine
DCC: *N,N*^′^-dicyclohexylcarbodiimide
PBS: phosphate-buffered saline
TREG: triethylene glycol

## References

[ref1] Arbyn M.; Weiderpass E.; Bruni L.; de Sanjosé S.; Saraiya M.; Ferlay J.; Bray F. Estimates of incidence and mortality of cervical cancer in 2018: a worldwide analysis. 2020, 8 ( (2), ), e203–e191, 10.1016/S2214-109X(19)30482-6. PMC7025157 31812369

[ref2] Siegel R. L.; Miller K. D.; Fuchs H. E.; Jemal A. Cancer Statistics. Ca-Cancer J. Clin. 2021, 71, 7–33. 10.3322/caac.21708. 33433946

[ref3] LaVigne A. W.; Triedman S. A.; Randall T. C.; Trimble E. L.; Viswanathan A. N. Cervical cancer in low and middle income countries: addressing barriers to radiotherapy delivery. Gynecol. Oncol. 2017, 22, 20–16. 10.1016/j.gore.2017.08.004. PMC5602511 28948205

[ref4] Adiga D.; Eswaran S.; Pandey D.; Sharan K.; Kabekkodu S. P. Molecular landscape of recurrent cervical cancer. Crit. Rev. Oncol. Hematol. 2021, 157, 103178 10.1016/j.critrevonc.2020.103178. 33279812

[ref5] Paskeh M. D. A.; Mirzaei S.; Gholami M. H.; Zarrabi A.; Zabolian A.; Hashemi M.; Hushmandi K.; Ashrafizadeh M.; Aref A. R.; Samarghandian S. Cervical cancer progression is regulated by SOX transcription factors: Revealing signaling networks and therapeutic strategies. Biomed. Pharmacother. 2021, 144, 112335 10.1016/j.biopha.2021.112335. 34700233

[ref6] Rabiee N.; Ahmadi S.; Fatahi Y.; Rabiee M.; Bagherzadeh M.; Dinarvand R.; Bagheri B.; Zarrintaj P.; Saeb M. R.; Webster T. J. Nanotechnology-assisted microfluidic systems: from bench to bedside. Nanomedicine 2020, 16, 237–258. 10.2217/nnm-2020-0353 33501839

[ref7] Rahimnejad M.; Rabiee N.; Ahmadi S.; Jahangiri S.; Sajadi S. M.; Akhavan O.; Saeb M. R.; Kwon W.; Kim M.; Hahn S. K. Emerging phospholipid nanobiomaterials for biomedical applications to lab-on-a-chip, drug delivery, and cellular engineering. ACS Appl. Bio Mater. 2021, 4, 8110–8128. 10.1021/acsabm.1c00932. 35005915

[ref8] Jafari Z.; Bigham A.; Sadeghi S.; Dehdashti S. M.; Rabiee N.; Abedivash A.; Bagherzadeh M.; Nasseri B.; Karimi-Maleh H.; Sharifi E. Nanotechnology-Abetted Astaxanthin Formulations in Multimodel Therapeutic and Biomedical Applications. J. Med. Chem. 2021, 65, 2–36. 10.1021/acs.jmedchem.1c01144. 34919379 PMC8762669

[ref9] Rahimnejad M.; Nasrollahi Boroujeni N.; Jahangiri S.; Rabiee N.; Rabiee M.; Makvandi P.; Akhavan O.; Varma R. S. Prevascularized micro–/nano-sized spheroid/bead aggregates for vascular tissue engineering. Nano-Micro Lett. 2021, 13, 1–24. 10.1007/s40820-021-00697-1. PMC8374027 34409511

[ref10] Ashrafizadeh M.; Najafi M.; Makvandi P.; Zarrabi A.; Farkhondeh T.; Samarghandian S. J. J. Versatile role of curcumin and its derivatives in lung cancer therapy. J. Cell. Physiol. 2020, 235, 9241–9268. 10.1002/jcp.29819. 32519340

[ref11] Farkhondeh T.; Ashrafizadeh M.; Azimi-Nezhad M.; Samini F.; Aschenr M.; Samarghandian S. Curcumin Efficacy in a Serum/glucose Deprivation-induced Neuronal PC12 Injury Model. Curr. Mol. Pharmacol. 2021, 14, 1155–1146. 10.2174/1874467214666210203211312. PMC8329120 33538682

[ref12] Abadi A. J.; Mirzaei S.; Mahabady M. K.; Hashemi F.; Zabolian A.; Hashemi F.; Raee P.; Aghamiri S.; Ashrafizadeh M.; Aref A. R. Curcumin and its derivatives in cancer therapy: Potentiating antitumor activity of cisplatin and reducing side effects. Phytother. Res. 2021, 36, 189–213. 10.1002/ptr.7305. 34697839

[ref13] Shang H. S.; Chang C. H.; Chou Y. R.; Yeh M. Y.; Au M. K.; Lu H. F.; Chu Y. L.; Chou H. M.; Chou H. C.; Shih Y. L.; Chung J. G. Curcumin causes DNA damage and affects associated protein expression in HeLa human cervical cancer cells. Oncol. Rep. 2016, 36, 2207–2215. 10.3892/or.2016.5002. 27499229

[ref14] Wang T.; Wu X.; Al Rudaisat M.; Song Y.; Cheng H. Curcumin induces G2/M arrest and triggers autophagy, ROS generation and cell senescence in cervical cancer cells. J. Cancer 2020, 11, 6704–6715. 10.7150/jca.45176. 33046993 PMC7545669

[ref15] Thacker P. C.; Karunagaran D. Curcumin and emodin down-regulate TGF-β signaling pathway in human cervical cancer cells. PLoS One 2015, 10, e0120045 10.1371/journal.pone.0120045. 25786122 PMC4365016

[ref16] Ashrafizadeh M.; Zarrabi A.; Hashemi F.; Zabolian A.; Saleki H.; Bagherian M.; Azami N.; Bejandi A. K.; Hushmandi K.; Ang H. L. J. P. Polychemotherapy with curcumin and doxorubicin via biological nanoplatforms: enhancing antitumor activity. Pharmaceutics 2020, 12, 1084 10.3390/pharmaceutics12111084. PMC7697177 33187385

[ref17] Saeb M. R.; Rabiee N.; Mozafari M.; Mostafavi E. Metal-organic frameworks-based nanomaterials for drug delivery. Materials 2021, 14, 3652 10.3390/ma14133652. 34208958 PMC8269711

[ref18] Rabiee N.; Bagherzadeh M.; Kiani M.; Ghadiri A. M.; Zhang K.; Jin Z.; Ramakrishna S.; Shokouhimehr M. High gravity-assisted green synthesis of ZnO nanoparticles via Allium ursinum: Conjoining nanochemistry to neuroscience. Nano Express 2020, 1, 020025.

[ref19] Rabiee N.; Bagherzadeh M.; Kiani M.; Ghadiri A. M.; Etessamifar F.; Jaberizadeh A. H.; Shakeri A. Biosynthesis of copper oxide nanoparticles with potential biomedical applications. Int. J. Nanomed. 2020, 15, 3983 10.2147/IJN.S255398. PMC7294052 32606660

[ref20] Rabiee N.; Bagherzadeh M.; Kiani M.; Ghadiri A. M. Rosmarinus officinalis directed palladium nanoparticle synthesis: investigation of potential anti-bacterial, anti-fungal and Mizoroki-Heck catalytic activities. Adv. Powder Technol. 2020, 31, 1402–1411. 10.1016/j.apt.2020.01.024.

[ref21] Kiani M.; Rabiee N.; Bagherzadeh M.; Ghadiri A. M.; Fatahi Y.; Dinarvand R.; Webster T. J. High-gravity-assisted green synthesis of palladium nanoparticles: the flowering of nanomedicine. Nanomed.: Nanotechnol., Biol. Med. 2020, 30, 102297 10.1016/j.nano.2020.102297. 32931927

[ref22] Rabiee N.; Bagherzadeh M.; Heidarian Haris M.; Ghadiri A. M.; Matloubi Moghaddam F.; Fatahi Y.; Dinarvand R.; Jarahiyan A.; Ahmadi S.; Shokouhimehr M. Polymer-Coated NH2-UiO-66 for the Codelivery of DOX/pCRISPR. ACS Appl. Mater. Interfaces 2021, 13, 10796–10811. 10.1021/acsami.1c01460. 33621063

[ref23] Saeb M. R.; Rabiee N.; Seidi F.; Far B. F.; Bagherzadeh M.; Lima E. C.; Rabiee M. Green CoNi2S4/Porphyrin Decorated Carbon-based Nanocomposites for Genetic Materials Detection. J. Bioresour. Bioprod. 2021, 6, 215–222. 10.1016/j.jobab.2021.06.001.

[ref24] Rabiee N.; Bagherzadeh M.; Ghadiri A. M.; Fatahi Y.; Aldhaher A.; Makvandi P.; Dinarvand R.; Jouyandeh M.; Saeb M. R.; Mozafari M. Turning Toxic Nanomaterials into a Safe and Bioactive Nanocarrier for Co-delivery of DOX/pCRISPR. ACS Appl. Bio Mater. 2021, 4, 5336–5351. 10.1021/acsabm.1c00447. 35007014

[ref25] Rabiee N.; Bagherzadeh M.; Ghadiri A. M.; Kiani M.; Ahmadi S.; Jajarmi V.; Fatahi Y.; Aldhaher A.; Tahriri M.; Webster T. J. Calcium-based nanomaterials and their interrelation with chitosan: Optimization for pCRISPR delivery. J. Nanostruct. Chem. 2021, 1–14. 10.1007/s40097-021-00446-1. PMC8457547 34580605

[ref26] Rabiee N.; Bagherzadeh M.; Tavakolizadeh M.; Pourjavadi A.; Atarod M.; Webster T. J. Synthesis, characterization and mechanistic study of nano chitosan tetrazole as a novel and promising platform for CRISPR delivery. Int. J. Polym. Mater. Polym. Biomater. 2022, 71, 116–126. 10.1080/00914037.2020.1809405.

[ref27] Rabiee N.; Bagherzadeh M.; Ghadiri A. M.; Salehi G.; Fatahi Y.; Dinarvand R. ZnAl nano layered double hydroxides for dual functional CRISPR/Cas9 delivery and enhanced green fluorescence protein biosensor. Sci. Rep. 2020, 10, 1–15. 10.1038/s41598-020-77809-1. 33244160 PMC7693303

[ref28] Rabiee N.; Bagherzadeh M.; Ghadiri A. M.; Kiani M.; Aldhaher A.; Ramakrishna S.; Tahriri M.; Tayebi L.; Webster T. J. Green synthesis of ZnO NPs via Salvia hispanica: Evaluation of potential antioxidant, antibacterial, mammalian cell viability, H1N1 influenza virus inhibition and photocatalytic activities. J. Biomed. Nanotechnol. 2020, 16, 456–466. 10.1166/jbn.2020.2916. 32970978

[ref29] Rabiee N.; Ahmadi S.; Rabiee M.; Bagherzadeh M.; Vahabi H.; Jouyandeh M.; Saeb M. R. Green carbon-based nanocomposite biomaterials through the lens of microscopes. Emergent Mater. 2021, 1–7. 10.1007/s42247-021-00277-4.

[ref30] Rabiee N.; Fatahi Y.; Asadnia M.; Daneshgar H.; Kiani M.; Ghadiri A. M.; Atarod M.; Mashhadzadeh A. H.; Akhavan O.; Bagherzadeh M. Green porous benzamide-like nanomembranes for hazardous cations detection, separation, and concentration adjustment. J. Hazard. Mater. 2022, 423, 127130 10.1016/j.jhazmat.2021.127130. 34530276

[ref31] Bagherzadeh M.; Rabiee N.; Fatahi Y.; Dinarvand R. Zn-rich (GaN) 1– x (ZnO) x: a biomedical friend?. New J. Chem. 2021, 45, 4077–4089. 10.1039/D0NJ06310J.

[ref32] Rabiee N.; Rabiee M.; Sojdeh S.; Fatahi Y.; Dinarvand R.; Safarkhani M.; Ahmadi S.; Daneshgar H.; Radmanesh F.; Maghsoudi S. Porphyrin molecules decorated on metal–organic frameworks for multi-functional biomedical applications. Biomolecules 2021, 11, 1714 10.3390/biom11111714. 34827712 PMC8615380

[ref33] Saeb M. R.; Rabiee N.; Mozafari M.; Verpoort F.; Voskressensky L. G.; Luque R. Metal–Organic Frameworks (MOFs) for Cancer Therapy. Materials 2021, 14, 7277 10.3390/ma14237277. 34885431 PMC8658485

[ref34] Rabiee N.; Bagherzadeh M.; Ghasemi A.; Zare H.; Ahmadi S.; Fatahi Y.; Dinarvand R.; Rabiee M.; Ramakrishna S.; Shokouhimehr M. Point-of-use rapid detection of sars-cov-2: nanotechnology-enabled solutions for the COVID-19 pandemic. Int. J. Mol. Sci. 2020, 21, 5126 10.3390/ijms21145126. PMC7404277 32698479

[ref35] Ahmadi S.; Rabiee N.; Fatahi Y.; Hooshmand S. E.; Bagherzadeh M.; Rabiee M.; Jajarmi V.; Dinarvand R.; Habibzadeh S.; Saeb M. R. Green chemistry and coronavirus. Sustainable Chem. Pharm. 2021, 21, 100415 10.1016/j.scp.2021.100415. PMC7927595 33686371

[ref36] Rabiee N.; Rabiee M.; Bagherzadeh M.; Rezaei N. COVID-19 and picotechnology: potential opportunities. Med. Hypotheses 2020, 144, 109917 10.1016/j.mehy.2020.109917. 32505072 PMC7263242

[ref37] Chung S.; Revia R.; Zhang M. J. N. H. Iron oxide nanoparticles for immune cell labeling and cancer immunotherapy. Nanoscale Horiz. 2021, 6, 696–717. 10.1039/D1NH00179E. 34286791 PMC8496976

[ref38] Heydari Sheikh Hossein H.; Jabbari I.; Zarepour A.; Zarrabi A.; Ashrafizadeh M.; Taherian A.; Makvandi P. Functionalization of Magnetic Nanoparticles by Folate as Potential MRI Contrast Agent for Breast Cancer Diagnostics. Molecules 2020, 25, 4053 10.3390/molecules25184053. PMC7570917 32899812

[ref39] Khan S.; Setua S.; Kumari S.; Dan N.; Massey A.; Hafeez B. B.; Yallapu M. M.; Stiles Z. E.; Alabkaa A.; Yue J.; Ganju A.; Behrman S.; Jaggi M.; Chauhan S. C. Superparamagnetic iron oxide nanoparticles of curcumin enhance gemcitabine therapeutic response in pancreatic cancer. Biomaterials 2019, 208, 83–97. 10.1016/j.biomaterials.2019.04.005. 30999154 PMC13521448

[ref40] Elbialy N. S.; Aboushoushah S. F.; Alshammari W. W. Long-term biodistribution and toxicity of curcumin capped iron oxide nanoparticles after single-dose administration in mice. Life Sci. 2019, 230, 76–83. 10.1016/j.lfs.2019.05.048. 31128136

[ref41] Aboushoushah S.; Alshammari W.; Darwesh R.; Elbaily N. Toxicity and biodistribution assessment of curcumin-coated iron oxide nanoparticles: Multidose administration. Life Sci. 2021, 277, 119625 10.1016/j.lfs.2021.119625. 34015288

[ref42] Hooshmand S.; Hayat S. M. G.; Ghorbani A.; Khatami M.; Pakravanan K.; Darroudi M. Preparation and Applications of Superparamagnetic Iron Oxide Nanoparticles in Novel Drug Delivery Systems: An Overview Article. Curr. Med. Chem. 2021. 10.2174/0929867327666200123152006 31971104

[ref43] Jafari S.; Tavakoli M. B.; Zarrabi A. Lomustine loaded superparamagnetic iron oxide nanoparticles conjugated with folic acid for treatment of glioblastoma multiforma (GBM). Iranian Journal of Pharmaceutical Research: IJPR 2020, 19, 134 10.22037/IJPR.2020.1101032. PMC7667540 33224218

[ref44] Sun C.; Sze R.; Zhang M. Folic acid-PEG conjugated superparamagnetic nanoparticles for targeted cellular uptake and detection by MRI. Journal of Biomedical Materials Research Part A: An Official Journal of The Society for Biomaterials, The Japanese Society for Biomaterials, and The Australian Society for Biomaterials and the Korean Society for Biomaterials 2006, 78, 550–557. 10.1002/jbm.a.30781. 16736484

[ref45] Zhang Q.; Wang C.; Qiao L.; Yan H.; Liu K. Superparamagnetic iron oxide nanoparticles coated with a folate-conjugated polymer. J. Mater. Chem. 2009, 19, 8393–8402. 10.1039/B910439A.

[ref46] Krais A.; Wortmann L.; Hermanns L.; Feliu N.; Vahter M.; Stucky S.; Mathur S.; Fadeel B. Targeted uptake of folic acid-functionalized iron oxide nanoparticles by ovarian cancer cells in the presence but not in the absence of serum. Nanomed.: Nanotechnol., Biol. Med. 2014, 10, 1421–1431. 10.1016/j.nano.2014.01.006. 24491397

[ref47] Rabiee N.; Bagherzadeh M.; Ghadiri A. M.; Fatahi Y.; Baheiraei N.; Safarkhani M.; Aldhaher A.; Dinarvand R. Bio-multifunctional noncovalent porphyrin functionalized carbon-based nanocomposite. Sci. Rep. 2021, 11, 1–15. 10.1038/s41598-021-86119-z. 33758300 PMC7988124

[ref48] Rabiee N.; Yaraki M. T.; Garakani S. M.; Garakani S. M.; Ahmadi S.; Lajevardi A.; Bagherzadeh M.; Rabiee M.; Tayebi L.; Tahriri M. Recent advances in porphyrin-based nanocomposites for effective targeted imaging and therapy. Biomaterials 2020, 232, 119707 10.1016/j.biomaterials.2019.119707. 31874428 PMC7008091

[ref49] Nik A. B.; Zare H.; Razavi S.; Mohammadi H.; Ahmadi P. T.; Yazdani N.; Bayandori M.; Rabiee N.; Mobarakeh J. I. Smart drug delivery: Capping strategies for mesoporous silica nanoparticles. Microporous Mesoporous Mater. 2020, 299, 110115 10.1016/j.micromeso.2020.110115.

[ref50] Rabiee N.; Bagherzadeh M.; Ghadiri A. M.; Kiani M.; Fatahi Y.; Tavakolizadeh M.; Pourjavadi A.; Jouyandeh M.; Saeb M. R.; Mozafari M. Multifunctional 3D hierarchical bioactive green carbon-based nanocomposites. ACS Sustainable Chem. Eng. 2021, 9, 8706–8720. 10.1021/acssuschemeng.1c00781.

[ref51] Ghasemi A.; Rabiee N.; Ahmadi S.; Hashemzadeh S.; Lolasi F.; Bozorgomid M.; Kalbasi A.; Nasseri B.; Dezfuli A. S.; Aref A. R. Optical assays based on colloidal inorganic nanoparticles. Analyst 2018, 143, 3249–3283. 10.1039/C8AN00731D. 29924108 PMC6042520

[ref52] Nasseri B.; Kocum I. C.; Seymen C. M.; Rabiee N. Penetration depth in nanoparticles incorporated radiofrequency hyperthermia into the tissue: comprehensive study with histology and pathology observations. IET Nanobiotechnol. 2019, 13, 634–639. 31432798 10.1049/iet-nbt.2019.0066 PMC8676181

[ref53] Kumar M.; Singh G.; Arora V.; Mewar S.; Sharma U.; Jagannathan N.; Sapra S.; Dinda A. K.; Kharbanda S.; Singh H. Cellular interaction of folic acid conjugated superparamagnetic iron oxide nanoparticles and its use as contrast agent for targeted magnetic imaging of tumor cells. Int. J. Nanomed. 2012, 7, 3503 10.2147/IJN.S32694. PMC3405889 22848174

[ref54] Bonvin D.; Bastiaansen J. A.; Stuber M.; Hofmann H.; Ebersold M. M. Folic acid on iron oxide nanoparticles: platform with high potential for simultaneous targeting, MRI detection and hyperthermia treatment of lymph node metastases of prostate cancer. Dalton Trans. 2017, 46, 12692–12704. 10.1039/C7DT02139A. 28914298

[ref55] Huang Y. S.; Lu Y. J.; Chen J. P. Magnetic graphene oxide as a carrier for targeted delivery of chemotherapy drugs in cancer therapy. J. Magn. Magn. Mater. 2017, 427, 34–40. 10.1016/j.jmmm.2016.10.042.

[ref56] Akbarzadeh A.; Mikaeili H.; Zarghami N.; Mohammad R.; Barkhordari A.; Davaran S. Preparation and in vitro evaluation of doxorubicin-loaded Fe3O4 magnetic nanoparticles modified with biocompatible copolymers. Int. J. Nanomed. 2012, 7, 511 10.2147/IJN.S24326. PMC3273983 22334781

[ref57] Malik P.; Ameta R.; Singh M. Preparation and characterization of bionanoemulsions for improving and modulating the antioxidant efficacy of natural phenolic antioxidant curcumin. Chem.-Biol. Interact. 2014, 222, 77–86. 10.1016/j.cbi.2014.07.013. 25110318

[ref58] Angelopoulou A.; Kolokithas-Ntoukas A.; Fytas C.; Avgoustakis K. Folic acid-functionalized, condensed magnetic nanoparticles for targeted delivery of doxorubicin to tumor cancer cells overexpressing the folate receptor. ACS Omega 2019, 4, 22214–22227. 10.1021/acsomega.9b03594. 31891105 PMC6933766

[ref59] Deb A.; Vimala R. Camptothecin loaded graphene oxide nanoparticle functionalized with polyethylene glycol and folic acid for anticancer drug delivery. J. Drug Delivery Sci. Technol. 2018, 43, 333–342. 10.1016/j.jddst.2017.10.025.

[ref60] Vandghanooni S.; Eskandani M.; Barar J.; Omidi Y. Antisense LNA-loaded nanoparticles of star-shaped glucose-core PCL-PEG copolymer for enhanced inhibition of oncomiR-214 and nucleolin-mediated therapy of cisplatin-resistant ovarian cancer cells. Int. J. Pharm. 2020, 573, 118729 10.1016/j.ijpharm.2019.118729. 31705975

[ref61] Günday C.; Anand S.; Gencer H. B.; Munafò S.; Moroni L.; Fusco A.; Donnarumma G.; Ricci C.; Hatir P. C.; Türeli N. G. Ciprofloxacin-loaded polymeric nanoparticles incorporated electrospun fibers for drug delivery in tissue engineering applications. Drug Delivery Transl. Res. 2020, 10, 706–720. 10.1007/s13346-020-00736-1. 32100267

[ref62] Sadeghzadeh H.; Pilehvar-Soltanahmadi Y.; Akbarzadeh A.; Dariushnejad H.; Sanjarian F.; Zarghami N. The effects of nanoencapsulated curcumin-Fe3O4 on proliferation and hTERT gene expression in lung cancer cells. Anti-Cancer Agents Med. Chem. 2017, 17, 1363–1373. 10.2174/1871520617666170213115756. 28270067

[ref63] Kuang Y.; Zhang J.; Xiong M.; Zeng W.; Lin X.; Yi X.; Luo Y.; Yang M.; Li F.; Huang Q. A novel nanosystem realizing curcumin delivery based on Fe3O4@ carbon dots nanocomposite for Alzheimer’s disease therapy. Frontiers in bioengineering and biotechnology 2020, 1335. 10.3389/fbioe.2020.614906 PMC7744485 33344438

[ref64] Lucena G. N.; dos Santos C. C.; Pinto G. C.; Amantéa B. E.; Piazza R. D.; Jafelicci M. Jr.; Marques R. F. C. Drug Delivery and Magnetic Hyperthermia Based on Surface Engineering of Magnetic Nanoparticles. Magnetic Nanoparticles in Human Health and Medicine: Current Medical Applications and Alternative Therapy of Cancer 2021, 231–249. 10.1002/9781119754725.ch11.

[ref65] Kudr J.; Haddad Y.; Richtera L.; Heger Z.; Cernak M.; Adam V.; Zitka O. Magnetic nanoparticles: From design and synthesis to real world applications. Nanomaterials 2017, 7, 243 10.3390/nano7090243. PMC5618354 28850089

[ref66] Fakhimikabir H.; Tavakoli M. B.; Zarrabi A.; Amouheidari A.; Rahgozar S. The role of folic acid-conjugated polyglycerol coated iron oxide nanoparticles on radiosensitivity with clinical electron beam (6 MeV) on human cervical carcinoma cell line: in vitro study. J. Photochem. Photobiol., B 2018, 182, 71–76. 10.1016/j.jphotobiol.2018.03.023. 29626804

[ref67] Hardwick J.; Taylor J.; Mehta M.; Satija S.; Paudel K. R.; Hansbro P. M.; Chellappan D. K.; Bebawy M.; Dua K. Targeting cancer using curcumin encapsulated vesicular drug delivery systems. Curr. Pharm. Des. 2021, 27, 2–14. 10.2174/1381612826666200728151610. 32723255

[ref68] Ningombam G. S.; Chattopadhyay D.; Sarkar K.; Kalkura S. N.; Singh N. R. Luminescent water dispersible core-shell–(Y/Eu/Li) VO4@ APTES@ Folate and (Y/Eu/Li) VO4@ Fe3O4@ PEG nanocomposites: Biocompatibility and induction heating within the threshold alternating magnetic field. Colloids Surf., A 2021, 625, 126826 10.1016/j.colsurfa.2021.126826.

[ref69] de Sousa M.; Visani de Luna L. A.; Fonseca L. C.; Giorgio S.; Alves O. L. Folic-acid-functionalized graphene oxide nanocarrier: synthetic approaches, characterization, drug delivery study, and antitumor screening. ACS Appl. Nano Mater. 2018, 1, 922–932. 10.1021/acsanm.7b00324.

[ref70] Depan D.; Shah J.; Misra R. Controlled release of drug from folate-decorated and graphene mediated drug delivery system: synthesis, loading efficiency, and drug release response. Mater. Sci. Eng., C 2011, 31, 1305–1312. 10.1016/j.msec.2011.04.010.

[ref71] Yang H.; Wang N.; Yang R.; Zhang L. M.; Jiang X. Folic Acid Decorated β-Cyclodextrin-Based Poly (ε-Caprolactone)/Dextran Star Polymer with Disulfide Bond-Linker as Theranostic Nanoparticle for Tumor-Targeted MRI and Chemotherapy. Pharmaceutics 2021, 14, 52 10.3390/pharmaceutics14010052. 35056948 PMC8778171

[ref72] Ashrafizadeh M.; Najafi M.; Makvandi P.; Zarrabi A.; Farkhondeh T.; Samarghandian S. Versatile role of curcumin and its derivatives in lung cancer therapy. J. Cell. Physiol. 2020, 235, 9241–9268. 10.1002/jcp.29819. 32519340

[ref73] Zafar A.; Alruwaili N. K.; Imam S. S.; Alharbi K. S.; Afzal M.; Alotaibi N. H.; Yasir M.; Elmowafy M.; Alshehri S. Novel nanotechnology approaches for diagnosis and therapy of breast, ovarian and cervical cancer in female: A review. J. Drug Delivery Sci. Technol. 2021, 61, 102198 10.1016/j.jddst.2020.102198.

[ref74] Hong R.; Feng B.; Chen L.; Liu G.; Li H.; Zheng Y.; Wei D. Synthesis, characterization and MRI application of dextran-coated Fe3O4 magnetic nanoparticles. Biochem. Eng. J. 2008, 42, 290–300. 10.1016/j.bej.2008.07.009.

[ref75] Latha S.; Selvamani P.; Palanisamy S. B.; Govindaraj D. B. T.; Thangavelu P. Magnetic Nanoparticles: Role in Next Generation Nanomedicine. In Handbook of Research on Nano-Strategies for Combatting Antimicrobial Resistance and Cancer, IGI Global: 2021; pp. 337–369.

[ref76] Pourjavadi A.; Asgari S.; Hosseini S. H.; Akhlaghi M. Codelivery of hydrophobic and hydrophilic drugs by graphene-decorated magnetic dendrimers. Langmuir 2018, 34, 15304–15318. 10.1021/acs.langmuir.8b02710. 30424605

