# Correction to “Folic Acid-Adorned Curcumin-Loaded Iron Oxide Nanoparticles for Cervical Cancer”

**DOI:** 10.1021/acsabm.2c01014

**Published:** 2023-01-09

**Authors:** Marzieh Ramezani Farani, Maryam Azarian, Hamid Heydari Sheikh Hossein, Zohreh Abdolvahabi, Zahra Mohammadi Abgarmi, Arash Moradi, Seyyedeh Maedeh Mousavi, Milad Ashrafizadeh, Pooyan Makvandi, Mohammad Reza Saeb, Navid Rabiee

After much investigation, we realized that there is a flaw in Figures 2 and 5. However, this correction does not change and alter the results of this study. We certify that these changes do not affect the results and conclusion, and happened by mistake. The correct results are shown below.

**Figure 2:**
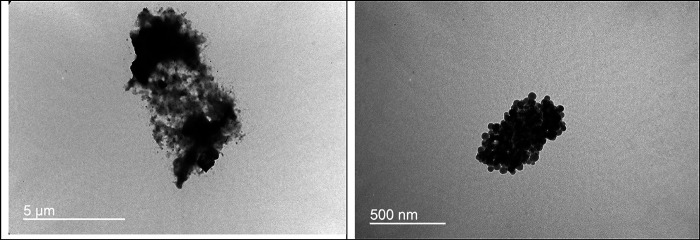
TEM images of (C, right panel) Fe_3_O_4_ and (D, left panel) HPG@Fe_3_O_4_ nanoparticles.

**Figure 5:**
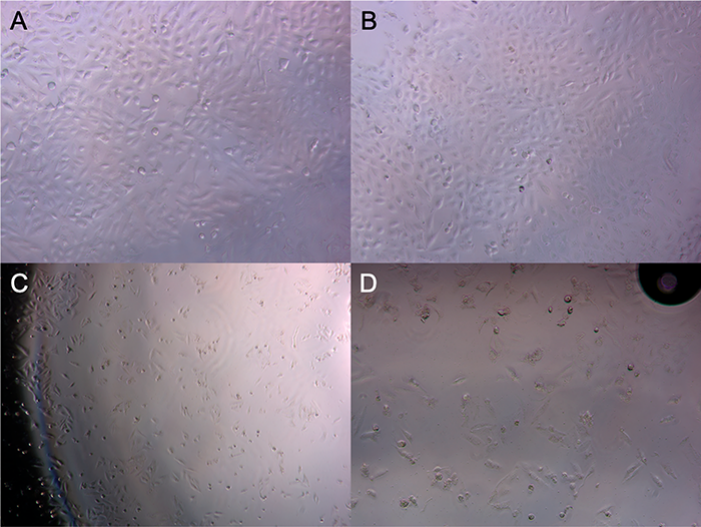
HeLa cell line’s optical microscopy images related to (A) before and (B) after MTT treatment. L929 cell line’s optical microscopy images related to (C) before and after (D) MTT.

The TEM and the cellular microscopy images were wrongly placed in Figures 2 and 5, respectively, and should be replaced with the new images.

